# Association of Diabetes With All-Cause and Cause-Specific Mortality in Asia: A Pooled Analysis of More Than 1 Million Participants

**DOI:** 10.1001/jamanetworkopen.2019.2696

**Published:** 2019-04-19

**Authors:** Jae Jeong Yang, Danxia Yu, Wanqing Wen, Eiko Saito, Shafiur Rahman, Xiao-Ou Shu, Yu Chen, Prakash C. Gupta, Dongfeng Gu, Shoichiro Tsugane, Yong-Bing Xiang, Yu-Tang Gao, Jian-Min Yuan, Akiko Tamakoshi, Fujiko Irie, Atsuko Sadakane, Yasutake Tomata, Seiki Kanemura, Ichiro Tsuji, Keitaro Matsuo, Chisato Nagata, Chien-Jen Chen, Woon-Puay Koh, Myung-Hee Shin, Sue K. Park, Pei-Ei Wu, You-Lin Qiao, Mangesh S. Pednekar, Jiang He, Norie Sawada, Hong-Lan Li, Jing Gao, Hui Cai, Renwei Wang, Toshimi Sairenchi, Eric Grant, Yumi Sugawara, Shu Zhang, Hidemi Ito, Keiko Wada, Chen-Yang Shen, Wen-Harn Pan, Yoon-Ok Ahn, San-Lin You, Jin-Hu Fan, Keun-Young Yoo, Habibul Ashan, Kee Seng Chia, Paolo Boffetta, Manami Inoue, Daehee Kang, John D. Potter, Wei Zheng

**Affiliations:** 1 Division of Epidemiology, Department of Medicine, Vanderbilt Epidemiology Center, Vanderbilt-Ingram Cancer Center, Vanderbilt University Medical Center, Nashville, Tennessee; 2 Division of Cancer Statistics Integration, Center for Cancer Control and Information Services, National Cancer Center, Tokyo, Japan; 3 Department of Global Health Policy, Graduate School of Medicine, University of Tokyo, Tokyo, Japan; 4 Department of Population Health, New York University School of Medicine, New York; 5 Department of Environmental Medicine, New York University School of Medicine, New York; 6 Healis-Sekhsaria Institute for Public Health, Mahape, Navi Mumbai, India; 7 Fuwai Hospital, National Center for Cardiovascular Diseases, Chinese Academy of Medical Sciences and Peking Union Medical College, Beijing, People’s Republic of China; 8 Epidemiology and Prevention Group, Center for Public Health Sciences, National Cancer Center, Tokyo, Japan; 9 State Key Laboratory of Oncogene and Related Genes, Shanghai Cancer Institute, Renji Hospital, Shanghai Jiaotong University School of Medicine, Shanghai, People’s Republic of China; 10 Department of Epidemiology, Shanghai Cancer Institute, Renji Hospital, Shanghai Jiaotong University School of Medicine, Shanghai, People’s Republic of China; 11 Graduate School of Public Health, University of Pittsburgh, Pittsburgh, Pennsylvania; 12 Graduate School of Medicine, Hokkaido University, Sapporo, Japan; 13 Department of Health and Welfare, Ibaraki Prefectural Office, Mito, Japan; 14 Radiation Effects Research Foundation, Hiroshima, Japan; 15 Tohoku University Graduate School of Medicine, Sendai, Japan; 16 Division of Molecular & Clinical Epidemiology, Aichi Cancer Center Research Institute, Nagoya, Japan; 17 Department of Epidemiology, Nagoya University Graduate School of Medicine, Nagoya, Japan; 18 Graduate School of Medicine, Gifu University, Gifu City, Japan; 19 Academia Sinica, Taipei City, Taiwan; 20 Health Services and Systems Research, Duke-NUS Medical School Singapore, Singapore, Republic of Singapore; 21 Saw Swee Hock School of Public Health, National University of Singapore, Singapore, Republic of Singapore; 22 Department of Social and Preventive Medicine, Sungkyunkwan University School of Medicine, Seoul, South Korea; 23 Department of Preventive Medicine, Seoul National University College of Medicine, Seoul, South Korea; 24 Department of Biomedical Sciences, Seoul National University Graduate School, Seoul, South Korea; 25 Cancer Research Institute, Seoul National University, Seoul, South Korea; 26 Taiwan Biobank, Institute of Biomedical Sciences, Academia Sinica, Taipei city, Taiwan; 27 National Cancer Center, Cancer Hospital, Chinese Academy of Medical Sciences, Beijing, People’s Republic of China; 28 Department of Epidemiology, Tulane University School of Public Health and Tropical Medicine, New Orleans, Louisiana; 29 Department of Public Health, Dokkyo Medical University School of Medicine, Mibu, Japan; 30 Institute of Biomedical Sciences, Academia Sinica, Taipei City, Taiwan; 31 College of Public Health, China Medical University, Taichung, Taiwan,; 32 School of Medicine & Big Data Research Center, Fu Jen Catholic University, Taipei City, Taiwan; 33 Armed Forces Capital Hospital, Seongnam, South Korea; 34 Department of Health Studies, University of Chicago, Chicago, Illinois; 35 Department of Medicine, University of Chicago, Chicago, Illinois; 36 Department of Human Genetics, University of Chicago, Chicago, Illinois; 37 Cancer Research Center, University of Chicago, Chicago, Illinois; 38 Icahn School of Medicine at Mount Sinai, New York, New York; 39 Division of Public Health Sciences, Fred Hutchinson Cancer Research Center, Seattle, Washington; 40 Centre for Public Health Research, Massey University, Wellington, New Zealand; 41 Department of Epidemiology, University of Washington, Seattle

## Abstract

**Importance:**

Asia is home to the largest diabetic populations in the world. However, limited studies have quantified the association of diabetes with all-cause and cause-specific mortality in Asian populations.

**Objectives:**

To evaluate the association of diabetes with all-cause and cause-specific mortality in Asia and to investigate potential effect modifications of the diabetes-mortality associations by participants’ age, sex, education level, body mass index, and smoking status.

**Design, Setting, and Participants:**

This pooled analysis incorporated individual participant data from 22 prospective cohort studies of the Asia Cohort Consortium conducted between 1963 and 2006. A total of 1 002 551 Asian individuals (from mainland China, Japan, South Korea, Singapore, Taiwan, India, and Bangladesh) were followed up for more than 3 years. Cohort-specific hazard ratios and 95% confidence intervals for all-cause and cause-specific mortality were estimated using Cox regression models and then pooled using random-effects meta-analysis. Analysis was conducted between January 10, 2018, and August 31, 2018.

**Exposures:**

Doctor-diagnosed diabetes, age, sex, education level, body mass index, and smoking status.

**Main Outcomes and Measures:**

All-cause and cause-specific mortality.

**Results:**

Of 1 002 551 participants (518 537 [51.7%] female; median [range] age, 54.0 [30.0-98.0] years), 148 868 deaths were ascertained during a median (range) follow-up of 12.6 (3.0-38.9) years. The overall prevalence of diabetes reported at baseline was 4.8% for men and 3.6% for women. Patients with diabetes had a 1.89-fold risk of all-cause death compared with patients without diabetes (hazard ratio [HR], 1.89; 95% CI, 1.74-2.04), with the highest relative risk of death due to diabetes itself (HR, 22.8; 95% CI, 18.5-28.1), followed by renal disease (HR, 3.08; 95% CI, 2.50-3.78), coronary heart disease (HR, 2.57; 95% CI, 2.19-3.02), and ischemic stroke (HR, 2.15; 95% CI, 1.85-2.51). The adverse diabetes-mortality associations were more evident among women (HR, 2.09; 95% CI, 1.89-2.32) than among men (HR, 1.74; 95% CI, 1.62-1.88) (*P* for interaction < .001) and more evident among adults aged 30 to 49 years (HR, 2.43; 95% CI, 2.08-2.84) than among adults aged 70 years and older (HR, 1.51; 95% CI, 1.40-1.62) (*P* for interaction < .001). A similar pattern of association was found between diabetes and cause-specific mortality, with significant variations noted by sex and age.

**Conclusions and Relevance:**

This study found that diabetes was associated with increased risk of death from several diseases among Asian populations. Development and implementation of diabetes management programs are urgently needed to reduce the burden of diabetes in Asia.

## Key Points

**Question:**

What is the association between diabetes and mortality in Asian populations?

**Findings:**

In this pooled analysis of data from more than 1 million individual participants of 22 studies in Asia, diabetes was associated with substantially increased risk of death from a broad spectrum of diseases, particularly diabetes itself, renal disease, coronary heart disease, and ischemic stroke. The associations were more evident among women and younger patients than among men and elderly patients.

**Meaning:**

The study’s findings suggest the urgent need for developing diabetes management programs that are tailored to Asian populations and the subsequent strong implementation of these programs in Asia.

## Teaser

This pooled analysis of data from more than 1 million participants examines the association of diabetes with all-cause and cause-specific mortality in Asian populations.

## Introduction

The global epidemic of diabetes has rapidly spread to every country in Asia.^1^ Currently, more than 230 million Asian individuals are living with diabetes, accounting for approximately 55% of the world’s diabetic population, and this number is expected to exceed 355 million by 2040.^2^ Two Asian countries, China and India, are home to the largest number of patients with diabetes in the world (110 million and 69.2 million, respectively).^2^ Three other Asian countries, Indonesia, Japan, and Bangladesh, are also ranked among the countries with the highest number of patients with diabetes worldwide.^1,2^ Given the increasing prevalence of obesity and the rapid adoption of a Westernized lifestyle in most Asian countries (especially developing countries),^3,4^ the diabetes epidemic will likely continue to impose a burden on public health systems across Asia.^1,3,5^

Emerging evidence suggests that diabetes in Asia shows unique features: compared with their Western counterparts, Asian individuals develop diabetes at a younger age and at a lower body mass index (BMI) and have a greater risk of developing complications, which may cause premature death.^1,5^ In addition, there are differences in genetics, socioeconomic factors, and diabetes management approaches between Asia and other continents, which may lead to a varied influence of diabetes on mortality in Asia.^1,6,7^ However, limited studies have prospectively quantified the association of diabetes with all-cause and cause-specific mortality in Asian populations and/or have examined potential effect modifiers on the association. In this pooled analysis of 22 prospective cohort studies, we assessed the association of diabetes with all-cause and cause-specific mortality in more than 1 million Asian individuals from multiple countries and regions and further investigated potential effect modifications of diabetes-mortality associations by participants’ age, sex, education level, BMI, and smoking status.

## Methods

### Study Populations

This study includes 22 prospective cohort studies in multiple countries and regions—mainland China, Japan, South Korea, Singapore, Taiwan, India, and Bangladesh—participating in the Asia Cohort Consortium (ACC). Details of the ACC have been described elsewhere.^8^ Brief descriptions of participating cohorts are given in the eAppendix in the Supplement. All cohort studies and the current pooled analysis received approval from the ethics committees of the host institutions. All study participants provided informed consent to the parent cohort studies. Each cohort collected baseline and outcome data according to its study protocol. The ACC coordinating center obtained deidentified individual participant data from each cohort and harmonized it across cohorts. This study follows the Strengthening the Reporting of Observational Studies in Epidemiology (STROBE) reporting guideline.

### Exposure and Outcome Assessment

Data on self-reported doctor-diagnosed diabetes were collected at baseline in all participating cohorts and used in the previous ACC pooled analyses.^8,9,10^ The overall validity of self-reported diabetes was evaluated in several participating cohorts, showing a very high specificity (approximately 99%) and a moderate sensitivity (approximately 62%).^9,10,11,12,13^ Information on types of diabetes was unavailable in the ACC; however, among one-third of patients with diabetes who reported their age at the first diagnosis, only 0.2% were diagnosed before the age of 20 years, which was consistent with a low prevalence of type 1 diabetes in Asia.^14^ Additionally, we excluded participants younger than 30 years at enrollment. Thus, nearly all patients with diabetes were diagnosed with type 2 diabetes. No clinical information, such as glucose levels and use of medications, was collected in most of the participating cohorts.

For mortality information, all study participants were followed up through data linkages to death certificates and/or active follow-up surveys. According to the *International Classification of Diseases, Ninth Revision* or *International Classification of Diseases, Tenth Revision,* the primary cause of death was coded for cause-specific mortality (eTable 1 in the Supplement). The Ibaraki Prefectural Health Study (Japan) ascertained the date of death without underlying causes; hence, it was only included in the analysis of all-cause mortality.

### Statistical Analysis

Of the 1 154 333 individuals included in these cohorts, we excluded 94 460 with unknown status on diabetes at baseline and 7889 with missing or incorrect information concerning vital status, duration of follow-up, or age at baseline (<30 years). To minimize the potential influence of underlying diseases other than diabetes on mortality risk, we further excluded data from the first 3 years of follow-up, including 49 433 participants who were followed up for less than 3 years. After these exclusions, 1 002 551 participants (484 014 men and 518 537 women) remained for the current analysis (Table 1).

**Table 1.** Characteristics of Participating Cohorts in the Asia Cohort Consortium

Cohort	Participants, No.^a^	Baseline Survey Dates	Follow-up, Median (Range), y^b^	Women, No. (%)	Age at Baseline, Median (Range), y	Diabetes, No. (%)		Deaths, No.
						Men	Women	
Mainland China								
China National Hypertension Survey Epidemiology Follow-up Study	138 640	1990-1992	8.2 (3.0-9.9)	70 737 (51.0)	54.0 (40.0-91.0)	1400 (2.1)	1515 (2.1)	12 453
Shanghai Cohort Study	17 703	1986-1989	18.0 (3.0-21.1)	NA	55.0 (35.0-76.0)	222 (1.3)	NA	4607
Shanghai Men’s Health Study	60 379	2001-2006	9.7 (3.0-13.4)	NA	52.0 (40.0-75.0)	3692 (6.1)	NA	4346
Shanghai Women’s Health Study	74 188	1996-2000	15.3 (3.0-18.4)	74 188 (100.0)	50.0 (40.0-70.0)	NA	3172 (4.3)	6903
Linxian General Population Trial Cohort	28 532	1984-1987	23.2 (3.0-25.8)	15 929 (55.8)	51.0 (30.0-81.0)	18 (0.1)	13 (0.1)	15 320
Japan								
3 Prefecture Aichi Study	20 529	1985-1985	15.2 (3.0-15.5)	11 048 (53.8)	53.0 (40.0-97.0)	554 (5.8)	368 (3.3)	2867
Ibaraki Prefectural Health Study	95 537	1993-1994	12.3 (3.0-12.7)	63 222 (66.2)	60.0 (39.0-79.0)	1255 (3.9)	1416 (2.2)	9721
Japan Collaborative Cohort Study	73 351	1988-1990	14.3 (3.0-16.0)	42 820 (58.4)	57.0 (40.0-79.0)	2060 (6.8)	1833 (4.3)	9387
Japan Public Health Center-Based prospective Study 1	42 548	1990-1992	22.6 (3.0-23.0)	22 255 (52.3)	50.0 (40.0-59.0)	1114 (5.5)	555 (2.5)	7069
Japan Public Health Center-Based Prospective Study 2	55 530	1992-1995	19.7 (3.0-20.0)	29 415 (53.0)	55.0 (40.0-69.0)	1980 (7.6)	1096 (3.7)	11 964
3 Prefecture Miyagi Study	27 954	1984-1984	15.0 (3.0-15.0)	15 679 (56.1)	56.0 (40.0-98.0)	879 (7.2)	648 (4.1)	5436
Miyagi Cohort	46 543	1990-1990	17.8 (3.0-17.8)	24 311 (52.2)	53.0 (40.0-64.0)	1182 (5.3)	771 (3.2)	5340
Ohsaki National Health Insurance Cohort Study	45 217	1994-1994	13.2 (3.0-13.2)	23 561 (52.1)	62.0 (40.0-80.0)	1568 (7.2)	1357 (5.8)	8141
Life Span Study	39 314	1963-1993	21.9 (3.0-38.9)	20 994 (53.4)	51.0 (30.0-97.0)	1681 (9.2)	1181 (5.6)	20 073
Takayama Study	25 694	1992-1992	15.6 (3.0-15.6)	14 217 (55.3)	54.0 (35.0-97.0)	840 (7.3)	471 (3.3)	4810
Korea								
Korean Multi-center Cancer Cohort Study	12 607	1993-2004	14.4 (3.0-20.5)	7665 (60.8)	56.0 (30.0-89.0)	222 (4.5)	360 (4.7)	2260
Seoul Male Cancer Cohort	13 767	1992-1993	16.0 (3.1-16.0)	NA	49.0 (31.0-82.0)	423 (3.1)	NA	836
Singapore								
Singapore Chinese Health Study	61 298	1993-1999	12.5 (3.0-14.9)	34 494 (56.3)	55.0 (43.0-83.0)	2161 (8.1)	3041 (8.8)	8733
Taiwan								
Community-Based Cancer Screening Project	23 425	1991-1992	15.9 (3.0-16.9)	11 703 (50.0)	47.0 (30.0-66.0)	318 (2.7)	251 (2.1)	2452
Cardiovascular Disease Risk Factor Two-Township Study	4264	1990-1993	15.7 (3.0-17.2)	2381 (55.8)	51.0 (30.0-91.0)	71 (3.8)	100 (4.2)	741
Bangladesh								
Health Effects for Arsenic Longitudinal Study	8489	2000-2002	12.7 (3.0-13.5)	4185 (49.3)	40.0 (30.0-75.0)	132 (3.1)	121 (2.9)	677
India								
Mumbai Cohort Study	87 042	1991-1997	5.7 (3.0-8.1)	29 733 (34.2)	50.0 (35.0-98.0)	1511 (2.6)	385 (1.3)	4732
Total	1 002 551	1963-2006	12.6 (3.0-38.9)	518 537 (51.7)	54.0 (30.0-98.0)	23 283 (4.8)	18 654 (3.6)	148 868

Abbreviation: NA, not available.

^a^ Includes only participants who were eligible for the current pooled analysis.

^b^ Median years of total follow-up time interval from study entry to the final date of last contact (end of follow-up, date of death, or loss to follow-up).

A 2-stage individual participant data meta-analysis was applied.^15^ First, we evaluated the associations between diabetes and mortality in each cohort separately. Then, cohort-specific hazard ratios (HRs) and 95% confidence intervals were pooled using the random-effects meta-analysis method.^16^ Proportionality assumption was tested using the global goodness-of-fit test with Schoenfeld residuals in each cohort—there was no evidence of a violation. In the Cox models, age was treated as the time scale, using age at enrollment (entry) and age at the final follow-up (exit), which was either the date of death or the date of last contact (ie, end of follow-up or loss to follow-up), whichever came first. All Cox models were stratified by 5-year intervals of birth year and enrollment year to account for the difference in calendar time across study populations. Covariates included age at baseline (continuous), smoking status (current, former, and never), smoking pack-year (0, 0.1-20.0, 20.1-40.0, and >40.0 pack-years), educational attainment (no formal schooling, primary education, secondary education, college education, university graduate, and postgraduate), marital status (single, married, and other), residential area (urban and rural), and BMI (<18.50, 18.50-24.99, 25.00-29.99, and ≥30.00; calculated as weight in kilograms divided by height in meters squared). The proportion of missing covariates was less than 10% in most participating cohorts. Missing covariates were imputed using the median (for continuous variables) or mode (for categorical variables) values of cohort-specific nonmissing covariates. In the sensitivity analyses, we excluded participants who had a history of cardiovascular disease (CVD) or cancer (except for nonmelanoma skin cancer) at baseline. Furthermore, we excluded 1 country at a time from the meta-analysis to assess the robustness of the overall findings.

To estimate the overall risk of death associated with diabetes among Asian populations, we first compared cause-specific death rates between individuals with and without diabetes. Death rates by diabetes status were estimated with age- and sex-specific deaths and person-years at risk, which were standardized according to the age and sex distribution of the ACC populations. The diabetes-mortality association, measured using HRs and 95% CIs, was evaluated for all participants and by sex (men and women), age at baseline (30-49, 50-59, 60-69, and ≥70 years), educational attainment (less than primary school graduation, secondary school, trade or technical school, and university graduation or greater), BMI (<18.50, 18.50-22.99, 23.00-24.99, and ≥25.00), and smoking status (never, former, and current). The interaction between diabetes and individual characteristics was tested by a 2-stage method^17^: the interactions were estimated in each cohort by using the likelihood ratio test entering a cross-product term of diabetes and the stratification variable. Then, the cohort-specific estimates (regression coefficients and variances) were pooled using random-effects meta-analysis. A 2-sided *P* value of less than .05 was considered statistically significant. All statistical procedures were conducted using SAS statistical software version 9.3 (SAS Institute Inc).

## Results

During a median (range) follow-up of 12.6 (3.0-38.9) years, 148 868 deaths were ascertained among 1 002 551 participants (518 537 [51.7%] female; median [range] age, 54.0 [30.0-98.0] years). The overall prevalence of self-reported diabetes was 4.8% for men and 3.6% for women (Table 1).

Diabetes was associated with a 1.89-fold increase in risk of death from all causes (HR, 1.89; 95% CI, 1.74-2.04) (Table 2). Specifically, patients with diabetes were at an increased risk of death from a wide range of diseases, with the highest relative risk for death due to diabetes itself (HR, 22.8; 95% CI, 18.5-28.1); followed by renal disease (HR, 3.08; 95% CI 2.50-3.78), including kidney failure (HR, 3.32; 95% CI, 2.67-4.12) and other renal disease (HR, 2.72; 95% CI, 1.98-3.74); coronary heart disease (HR, 2.57; 95% CI, 2.19-3.02); tuberculosis (HR, 2.28; 95% CI, 1.41-3.69); liver disorder (HR, 2.24; 95% CI, 1.87-2.68); and ischemic stroke (HR, 2.15; 95% CI, 1.85-2.51). Significant positive associations with diabetes were also found for risk of death from hemorrhagic stroke, digestive system disease, certain cancers (eg, liver, breast, pancreas, gallbladder, and colon or rectum), infectious disease, and mental disorder. However, a significant inverse association was found between diabetes and death from chronic obstructive pulmonary disease.

**Table 2.** All-Cause and Cause-Specific Mortality Associated With Diabetes in Asian Populations

Cause	Diabetes		No Diabetes		Adjusted HR (95% CI)^b^
	Deaths, No.	Death Rate per 1000 Person-Years^a^	Deaths, No.	Death Rate per 1000 Person-Years^a^	
All causes	12 465	166	136 403	126.4	1.89 (1.74-2.04)
Cardiovascular disease					
Coronary heart disease	1471	17.4	9107	6.8	2.57 (2.19-3.02)
Stroke, all types	1582	20.4	16 235	17.1	1.81 (1.61-2.04)
Ischemic stroke	576	7.4	4815	7.4	2.15 (1.85-2.51)
Hemorrhage stroke	470	7.4	6433	5	1.38 (1.17-1.62)
Other	1094	15.7	12 181	16.5	1.73 (1.53-1.96)
Endocrine, nutritional, and metabolic disease					
Diabetes	1456	17.1	1165	0.6	22.8 (18.5-28.1)
Other	31	1.2	681	0.6	1.68 (1.08-2.61)
Cancer					
Lung	501	5.2	8380	4.8	0.98 (0.89-1.07)
Breast	76	0.9	1071	0.9	1.74 (1.28-2.38)
Colorectal	359	3.3	4274	2.9	1.30 (1.16-1.46)
Stomach	394	4.8	6880	4.2	1.10 (0.96-1.25)
Liver	459	4.9	4067	2.5	1.89 (1.59-2.24)
Pancreas	224	2.6	2393	1.3	1.55 (1.31-1.84)
Gallbladder	136	1.4	1628	1	1.38 (1.16-1.65)
Female reproductive organs^c^	67	0.9	1160	0.7	1.21 (0.93-1.56)
Other	723	8.7	10 430	7.4	1.22 (1.13-1.32)
Renal disease					
Kidney failure	232	2.7	1211	1.5	3.32 (2.67-4.12)
Other	181	2.6	1104	0.8	2.72 (1.98-3.74)
Digestive system disease					
Liver disorder^d^	227	3.7	2234	1.8	2.24 (1.87-2.68)
Gallbladder, biliary tract, and pancreas	45	0.5	452	2.8	2.04 (1.48-2.80)
Stomach, esophagus, and duodenum	34	0.3	402	0.5	1.73 (1.20-2.49)
Other	131	1.3	1208	1.1	2.10 (1.63-2.70)
Respiratory disease					
Pneumonia	790	18.8	6595	11.9	1.79 (1.51-2.11)
Asthma	27	0.3	581	0.6	1.20 (0.81-1.79)
Chronic obstructive pulmonary disease	100	0.7	2549	1.8	0.78 (0.63-0.96)
Other	232	2.2	2,336	2.8	1.59 (1.38-1.82)
Infectious disease					
Tuberculosis	38	0.5	802	0.9	2.28 (1.41-3.69)
Other	162	8.5	1701	1.1	2.00 (1.62-2.47)
Other known disease					
Mental disorder	32	0.4	542	0.7	1.93 (1.22-3.05)
Nervous system disorder	63	0.6	1193	1	1.10 (0.84-1.45)
Other	697	10.4	10 060	16.5	1.42 (1.23-1.64)
Not specified^e^	901	7.8	23 781	13	1.73 (1.61-1.85)

Abbreviation: HR, hazard ratio.

^a^ Death rate per 1000 person-years, standardized by age and sex.

^b^ Adjusted for age, sex, smoking status, smoking pack-years, education, marital status, rural vs urban residence, and obesity status and stratified by birth year (5-year groups) and enrollment year (5-year groups).

^c^ Malignant neoplasms of vagina, cervix uteri, corpus uteri, uterus, ovary, vulva, and other female genital organs.

^d^ Liver cirrhosis, hepatic failure, chronic hepatitis, fibrosis, alcoholic or toxic liver disease, and other kinds of liver disorders.

^e^ Unknown causes including missing and invalid data on cause of death.

We found some differences in the diabetes-mortality association by country and region: the relative risks were greater than 2.6-fold in Taiwan and rural China; approximately 2.2-fold in Bangladesh, Singapore, and urban China; and less than 2.0-fold in Japan, South Korea, and India (*P* for heterogeneity <.001) (eFigure in the Supplement). Furthermore, the magnitude of the diabetes-mortality association differed by sex, age, and smoking status among women (Table 3). The relative risk for all-cause mortality was greater among women (HR, 2.09; 95% CI, 1.89-2.32) than among men (HR, 1.74; 95% CI, 1.62-1.88) (*P* for interaction < .001) and greater among participants aged 30 to 49 years (HR, 2.43; 95% CI, 2.08-2.84) than for those aged 50 to 59 years (HR, 2.06; 95% CI, 1.83-2.31), 60 to 69 years (HR, 1.87; 95% CI, 1.71-2.05), and 70 years or older (HR, 1.51; 95% CI, 1.40-1.62) (*P* for interaction <.001). Among women only, the diabetes-mortality association was stronger among those who had never smoked than among current smokers (HR, 2.13; 95% CI, 1.93-2.36 vs HR, 1.81; 95% CI, 1.59-2.06, respectively) (*P* for interaction = .009). No significant differences were found by smoking status among men, educational attainment, or BMI levels.

**Table 3.** All-Cause Mortality Associated With Diabetes Stratified by Individual Characteristics in Asian Populations

Characteristic	No.				Adjusted HR (95% CI)^a^	*P* Value for Interaction
	Diabetes		No Diabetes			
	Participants	Death	Participants	Death		
Sex						
Men	23 283	7231	460 731	79 405	1.74 (1.62-1.88)	<.001
Women	18 654	5234	499 883	56 998	2.09 (1.89-2.32)	
Age at baseline, y						
<50	7386	1106	359 919	19 086	2.43 (2.08-2.84)	<.001
50-59	13 742	3131	303 771	37 104	2.06 (1.83-2.31)	
60-69	15 262	5372	222 688	51 069	1.87 (1.71-2.05)	
≥70	5547	2856	74 236	29 144	1.51 (1.40-1.62)	
Educational attainment^b^						
≤Primary school	15 392	5410	326 333	65 069	1.98 (1.74-2.24)	.72
Secondary school	10 584	2820	228 826	27 315	1.94 (1.74-2.17)	
Trade or technical school	3814	764	99 961	6687	1.79 (1.65-1.95)	
≥University graduation	3369	655	102 115	7900	1.69 (1.47-1.94)	
Body mass index^c^						
<18.5	1500	687	69 637	14 564	1.87 (1.62-2.15)	.24
18.5-22.9	14 813	4847	427 259	64 845	1.96 (1.77-2.16)	
23.0-24.9	10 913	3127	224 093	28 238	1.86 (1.69-2.04)	
≥25.0	14 711	3804	239 625	28 756	1.85 (1.69-2.02)	
Smoking status for men						
Never	6799	1593	151 267	18 616	1.84 (1.64-2.08)	.14
Former	5237	1778	67 465	13 317	1.62 (1.51-1.75)	
Current	11 247	3860	241 999	47 472	1.70 (1.58-1.83)	
Smoking status for women						
Never	16 749	4514	462 968	50 414	2.13 (1.93-2.36)	.009
Former	451	177	5652	1095	1.98 (1.55-2.52)	
Current	1454	543	31 263	5489	1.81 (1.59-2.06)	

Abbreviation: HR, hazard ratio.

^a^ Adjusted for age, sex, smoking status, smoking pack-years, education, marital status, rural vs urban residence, and obesity status and stratified by birth year (5-year groups) and enrollment year (5-year groups).

^b^ Cohorts (3 Prefecture Aichi Study, 3 Prefecture Miyagi Study, Ibaraki Prefectural Health Study, Japan Public Health Center-Based Prospective Study 2, Korean Multi-center Cancer Cohort Study) without information on education were not included in the analysis.

^c^ Calculated as weight in kilograms divided by height in meters squared.

Potential modifying effects of sex, age, and BMI on the associations of diabetes with cause-specific mortality in Asian populations are shown in Table 4 and Table 5 and eTable 2 in the Supplement. Multiple CVD mortality outcomes (ie, total CVD, coronary heart disease, and ischemic stroke) and renal disease mortality showed stronger associations with diabetes among women than among men. Meanwhile, many mortality outcomes, including deaths due to CVD, digestive disease, respiratory disease, and infectious disease, showed stronger associations with diabetes among younger than among elderly adults. Moreover, the association of diabetes with risk of death due to diabetes itself was much stronger among those with a BMI less than 18.5 (HR, 95.4; 95% CI, 38.0-239.8) than among those with a BMI of 25.0 or greater (HR, 13.2; 95% CI, 10.7-16.4) (*P *for interaction < .001).

**Table 4.** Cause-Specific Mortality Associated With Diabetes Stratified by Individual Characteristics in Asian Populations: Death from Cardiometabolic Diseases

Characteristic	Adjusted HR (95% CI)^a^				
	Cardiovascular Disease	Coronary Heart Disease	Ischemic Stroke	Hemorrhagic Stroke	Diabetes Mellitus
All patients	2.00 (1.76-2.29)	2.57 (2.19-3.02)	2.15 (1.85-2.51)	1.38 (1.17-1.62)	22.8 (18.5-28.1)
Sex					
Men	1.82 (1.58-2.09)	2.22 (1.86-2.64)	1.74 (1.49-2.04)	1.35 (1.12-1.62)	23.0 (17.6-30.0)
Women	2.30 (1.96-2.71)	3.25 (2.64-4.00)	2.95 (2.29-3.78)	1.41 (1.17-1.68)	20.5 (16.3-25.8)
* P* value for interaction	<.001	<.001	.002	.74	.54
Age at baseline, y					
<50	2.95 (2.21-3.92)	3.36 (2.29-4.94)	5.62 (2.37-13.4)	2.42 (1.67-3.49)	29.0 (18.5-45.5)
50-59	2.43 (2.00-2.96)	3.04 (2.43-3.80)	3.09 (2.32-4.13)	1.58 (1.24-2.00)	21.3 (16.4-27.7)
60-69	1.96 (1.70-2.27)	2.64 (2.15-3.23)	2.05 (1.75-2.40)	1.45 (1.20-1.75)	20.3 (15.2-27.1)
≥70	1.46 (1.30-1.64)	1.92 (1.60-2.29)	1.66 (1.41-1.95)	0.95 (0.74-1.23)	21.7 (15.6-30.2)
* P* value for interaction	<.001	<.001	<.001	<.001	.10
Educational attainment^b^					
≤Primary school	2.01 (1.64-2.45)	2.84 (2.25-3.60)	2.15 (1.74-2.66)	1.24 (1.02-1.51)	19.4 (15.5-24.3)
Secondary school	2.05 (1.75-2.39)	2.47 (2.01-3.03)	2.69 (1.96-3.69)	1.55 (1.25-1.92)	26.9 (19.3-37.7)
Trade or technical school	2.12 (1.87-2.42)	2.56 (2.03-3.23)	1.91 (1.03-3.53)	2.00 (1.42-2.80)	23.1 (12.6-42.5)
≥University graduation	2.00 (1.57-2.56)	2.26 (1.51-3.36)	1.76 (1.04-2.99)	2.04 (1.34-3.11)	19.6 (12.2-31.5)
* P* value for interaction	.16	.16	.28	.01	.10
Body mass index^c^					
<18.5	1.76 (1.41-2.20)	2.51 (1.65-3.82)	1.45 (0.66-3.17)	1.39 (0.85-2.26)	95.4 (38.0-239.8)
18.5-22.9	2.09 (1.79-2.44)	2.71 (2.26-3.26)	2.07 (1.73-2.48)	1.59 (1.36-1.86)	33.5 (24.6-45.7)
23.0-24.9	2.11 (1.84-2.43)	2.65 (2.19-3.21)	2.56 (2.04-3.21)	1.64 (1.26-2.13)	22.1 (18.3-26.7)
≥25.0	1.89 (1.61-2.21)	2.47 (2.02-3.01)	2.21 (1.78-2.75)	1.20 (0.86-1.66)	13.2 (10.7-16.4)
* P* value for interaction	.20	.16	.39	.07	<.001
Smoking status for men					
Never	2.04 (1.67-2.49)	2.69 (2.08-3.46)	1.76 (1.14-2.71)	1.38 (0.94-2.02)	25.8 (18.2-36.4)
Former	1.67 (1.37-2.05)	1.98 (1.52-2.59)	1.87 (1.45-2.42)	1.69 (1.27-2.23)	23.9 (15.6-36.7)
Current	1.81 (1.58-2.07)	2.24 (1.89-2.65)	1.81 (1.52-2.16)	1.41 (1.19-1.68)	21.1 (15.5-28.9)
* P* value for interaction	.63	.19	.59	.72	.19
Smoking status for women					
Never	2.35 (1.99-2.78)	3.34 (2.73-4.08)	2.83 (2.21-3.62)	1.42 (1.17-1.73)	20.4 (16.3-25.5)
Former or current^d^	1.97 (1.55-2.51)	2.91 (2.06-4.12)	3.12 (1.83-5.32)	1.48 (1.03-2.12)	15.1 (10.0-22.9)
* P* value for interaction	.48	.14	.37	.78	.17

Abbreviation: HR, hazard ratio.

^a^ Adjusted for age, sex, smoking status, smoking pack-years, education, marital status, rural vs urban residence, and obesity status and stratified by birth year (5-year groups) and enrollment year (5-year groups).

^b^ Cohorts (3 Prefecture Aichi Study, 3 Prefecture Miyagi Study, Ibaraki Prefectural Health Study, Japan Public Health Center-Based Prospective Study 2, Korean Multi-center Cancer Cohort Study) without information on education were not included in the analysis.

^c^ Calculated as weight in kilograms divided by height in meters squared.

^d^ Former and current smokers were collapsed into 1 category to improve the stability of the point estimate.

**Table 5.** Cause-Specific Mortality Associated With Diabetes Stratified by Individual Characteristics in Asian Populations: Death From Other Major Diseases

Characteristic	Adjusted HR (95% CI)^a^				
	Total Cancer	Renal Disease	Digestive Disease	Respiratory Disease	Infectious Disease
All patients	1.21 (1.13-1.29)	3.08 (2.50-3.78)	1.98 (1.73-2.25)	1.45 (1.29-1.63)	1.96 (1.59-2.41)
Sex					
Men	1.20 (1.13-1.29)	2.85 (2.23-3.66)	2.02 (1.78-2.30)	1.50 (1.35-1.68)	1.95 (1.50-2.54)
Women	1.23 (1.12-1.36)	3.44 (2.66-4.45)	1.95 (1.63-2.33)	1.52 (1.25-1.83)	2.09 (1.62-2.70)
* P* value for interaction	.50	.01	.91	.53	.86
Age at baseline, y					
<50	1.24 (1.10-1.40)	9.65 (5.04-18.5)	2.90 (2.21-3.82)	3.60 (2.36-5.48)	3.66 (2.00-6.67)
50-59	1.30 (1.16-1.45)	5.09 (3.71-6.97)	2.53 (1.94-3.30)	2.00 (1.70-2.36)	2.68 (1.81-3.95)
60-69	1.18 (1.08-1.30)	2.85 (2.03-4.00)	1.85 (1.56-2.19)	1.50 (1.27-1.76)	1.88 (1.43-2.47)
≥70	1.18 (1.07-1.29)	2.10 (1.64-2.69)	1.52 (1.18-1.97)	1.18 (1.02-1.36)	2.32 (1.64-3.28)
* P* value for interaction	.11	.66	<.001	<.001	.04
Educational attainment^b^					
≤Primary school	1.20 (1.08-1.33)	3.44 (2.51-4.72)	1.83 (1.47-2.29)	1.41 (1.19-1.68)	2.20 (1.72-2.81)
Secondary school	1.28 (1.16-1.41)	3.57 (2.53-5.03)	2.40 (1.89-3.06)	1.75 (1.44-2.12)	2.29 (1.47-3.58)
Trade or technical school	1.27 (1.10-1.46)	3.19 (1.83-5.58)	2.97 (1.91-4.62)	1.69 (1.10-2.61)	2.14 (1.26-3.63)
≥University graduation	1.08 (0.91-1.28)	2.45 (0.87-6.91)	2.76 (1.70-4.49)	1.66 (0.96-2.87)	1.91 (0.67-5.46)
* P* value for interaction	.88	.81	.02	.47	.84
Body mass index^c^					
<18.5	1.36 (1.14-1.62)	4.29 (2.68-6.86)	2.80 (1.71-4.60)	1.24 (0.95-1.60)	2.22 (0.98-5.03)
18.5-22.9	1.25 (1.15-1.36)	3.59 (2.66-4.86)	2.07 (1.59-2.68)	1.48 (1.27-1.73)	2.07 (1.54-2.78)
23.0-24.9	1.20 (1.09-1.33)	2.91 (2.33-3.63)	2.26 (1.83-2.78)	1.66 (1.45-1.89)	2.42 (1.76-3.33)
≥25.0	1.23 (1.10-1.38)	3.09 (2.26-4.24)	2.04 (1.65-2.51)	1.64 (1.34-2.00)	2.20 (1.61-3.01)
* P* value for interaction	.95	.09	.37	.007	.49
Smoking status for men					
Never	1.18 (1.00-1.40)	3.48 (2.51-4.83)	2.31 (1.68-3.19)	1.71 (1.40-2.10)	2.55 (1.58-4.12)
Former	1.25 (1.13-1.38)	2.32 (1.64-3.28)	1.85 (1.33-2.58)	1.62 (1.42-1.85)	2.33 (1.61-3.35)
Current	1.19 (1.09-1.29)	2.90 (2.05-4.11)	2.45 (2.01-2.98)	1.47 (1.26-1.72)	2.02 (1.53-2.66)
* P* value for interaction	.39	.42	.18	.09	.55
Smoking status for women					
Never	1.27 (1.16-1.40)	3.46 (2.62-4.59)	1.84 (1.53-2.22)	1.59 (1.30-1.95)	2.17 (1.63-2.89)
Former or current^d^	1.07 (0.89-1.28)	3.65 (2.31-5.77)	2.82 (1.81-4.41)	1.47 (1.03-2.11)	2.45 (1.09-5.50)
* P* value for interaction	.07	.19	.03	.23	.54

Abbreviation: HR, hazard ratio.

^a^ Adjusted for age, sex, smoking status, smoking pack-years, education, marital status, rural vs urban residence, and obesity status and stratified by birth year (5-year groups) and enrollment year (5-year groups).

^b^ Cohorts (3 Prefecture Aichi Study, 3 Prefecture Miyagi Study, Ibaraki Prefectural Health Study, Japan Public Health Center-Based Prospective Study2, Korean Multi-center Cancer Cohort Study) without information on education were not included in the analysis.

^c^ Calculated as weight in kilograms divided by height in meters squared.

^d^ Former and current smokers were collapsed into 1 category to improve the stability of the point estimate.

Sensitivity analyses showed that (1) the association between diabetes and mortality did not change when we excluded the participants who had a history of CVD or cancer (eTable 3 in the Supplement) and (2) our overall results were not driven by the results from any single cohort or country (eTable 4 in the Supplement).

## Discussion

This pooled analysis of more than 1 million individuals from 22 Asian cohort studies showed that diabetes is associated with substantially increased risk of death from several diseases, particularly diabetes itself, renal disease, coronary heart disease, and ischemic stroke. Moreover, the magnitude of the associations varied by sex, age, BMI, and smoking status. Women and middle-aged adults showed higher diabetes-related risk of death from all causes, total CVD, coronary heart disease, ischemic stroke, and renal disease than men and older adults. Among women, the risk for all-cause mortality associated with diabetes appeared stronger among those who had never smoked than among current or former smokers. The relative risk of death due to diabetes itself was much stronger among individuals who were underweight than among those who were overweight or obese.

Previous studies^18,19,20,21,22,23,24^ have reported that diabetes leads to an approximate 2-fold risk for all-cause mortality, but the magnitude of the association seems to vary by study. Generally, studies conducted in Western populations^20,21,23^ reported HRs of 1.15 to 1.90 for all-cause mortality associated with diabetes. Meanwhile, studies conducted in developing countries reported greater HRs, such as 2.00 in mainland China (1.83 in urban areas and 2.17 in rural areas)^18^ and 1.9 to 5.4 in Mexico.^19^ A recent meta-analysis^24^ including 709 503 participants from Western populations and 271 290 Asian individuals (mostly from South Korean) reported an HR for all-cause mortality of 1.69. Our study includes other Asian populations and has a much larger sample size, enabling us to assess associations of diabetes with a larger number of mortality outcomes and potential modifiers in a broader Asian population. The variation in the magnitude of the diabetes-mortality association may be attributed, in part, to differences in diabetes care across populations. Many Asian patients with diabetes, especially those living in less-developed areas, fail to achieve optimal glycemic control and vascular protection owing to a lack of access to health care, antidiabetic medications, and education on diabetes management.^1^

For cause-specific mortality, diabetes was associated with a high relative risk of death from diabetes itself in Asian populations. The excess risks observed in our study are exceptionally high compared with those observed in the United States and in other countries.^19,20,23^ Furthermore, Asian diabetic populations were much more likely to die from renal disease, the major complication of diabetes, which is in line with the results from studies conducted in low-income countries.^18,25^ Evidence has indicated that Asian individuals are more susceptible to insulin resistance, early-onset type 2 diabetes and diabetes unrelated to obesity, and other metabolic disorders (eg, hypertension, hyperlipidemia, and visceral obesity) than populations with European ancestry.^1,5^ It is possible that these Asian-specific diabetes phenotypes are associated with a poor prognosis for diabetes and/or elevated risks of acute and chronic complications such as diabetic ketoacidosis and renal failure. Developing and implementing Asian-specific comprehensive diabetes management programs should help reduce mortality due to diabetes itself and/or its complications.

Consistent with the previous report from the ACC^10^ and other Asian studies,^22,26^ we observed that diabetes is associated with an increased risk of cancer mortality. In site-specific analyses, we found a positive association with liver, pancreas, and breast cancer, with an etiologic component that includes insulin resistance and obesity, which is in line with previous studies.^21,23,26,27,28^ Other findings for a positive association of diabetes with risk of death due to liver disorder; pancreas, gallbladder, and biliary tract-related disease; infection; and mental disorder are supported by previous studies.^18,19,21,22,23^ The pathogenesis of type 2 diabetes involves insulin resistance and impaired insulin secretion by β-cell dysfunction.^1,29^ Insulin secretion is modulated by the interplay of the pancreas with other organs and tissues such as liver, brain, digestive tracts, and adipose and muscle tissues.^30^ Our findings indicate an increased risk of death due to diseases of these insulin-related organs and tissues among patients with diabetes, highlighting the multifactorial and complicated effect of diabetes and the need for comprehensive health care strategies aimed at the effective management of diabetes and its complications.

We found that the association between diabetes and mortality in Asia varies by sex, age, and BMI, which is supported by previous findings.^19,20,21,22,23,24,31,32,33^ A previous study suggested that sex difference in the diabetes-mortality association may be attributed, in part, to sex hormones such as estrogens and androgens.^24^ It is also possible that the higher relative risk among women than among men, especially for CVD and renal disease mortality, may reflect gender inequality in diabetes care. Even in developed Western countries, female patients are less likely than male patients to receive optimal care and adhere to recommended treatment.^34^ Our findings for a stronger association of diabetes with risk of death (both all-cause and cause-specific) in younger populations than in elderly populations are also supported by previous studies.^19,20,24^ These findings are perhaps not surprising because early-onset type 2 diabetes (ie, onset before middle age) is more closely associated with macrovascular cardiovascular complications than late-onset diabetes.^35^ Of note, given the increasing prevalence of early-onset diabetes in Asia,^1,3,4^ the burden of premature death associated with diabetes is unfailingly projected to increase in the near future.^2^ In addition, the risk of diabetes mortality is much stronger among underweight individuals in Asian populations. Underweight patients may reflect the severity of diabetes and/or the presence of complications due to the poor management of the disease. The significant differences in the diabetes-mortality associations by individual characteristics highlight the need for further attention to vulnerable groups, such as women and young or underweight patients with diabetes, when implementing diabetes management plans at a population level.

### Strengths and Limitations 

To our knowledge, this is the largest prospective investigation of the impact of diabetes on all-cause and cause-specific mortality among Asian populations. In contrast to previous studies that were limited mostly to Asian individuals from developed countries,^22,24^ our study populations were composed of Asian individuals from multiple countries and regions at various levels of economic development. The large sample size and the availability of individual participant data of more than 1 million Asian individuals from 22 prospective cohort studies yields great statistical power to assess potential variations in the diabetes-mortality associations by participants’ characteristics.

However, we acknowledge several study limitations. First, data on diabetes diagnosis by their doctors were self-reported at baseline. It is possible that some patients who were not aware of their diabetes diagnosis at baseline or were diagnosed with diabetes during the cohort follow-up have been misclassified into the reference group, which could have attenuated the association. The validity of self-reported data in diabetes classification showed a sensitivity of approximately 62% and a specificity of approximately 99% in several participating cohorts.^9,10,11,12,13^ With a relatively low prevalence of diabetes in the Asian population, a low sensitivity has less impact on study results than a low specificity. Second, baseline surveys were conducted at different time periods (1963-2006); thus, our results may potentially be influenced by recent changes in diabetes diagnosis and treatment and lifestyle transitions in some Asian countries. To minimize potential time and period effects, our models were stratified by cohort enrollment year and participants’ birth year. Future studies using more recent data on diabetes diagnosis, treatment, and lifestyle factors could further evaluate the association of diabetes with mortality outcomes in Asia. Third, we cannot rule out potential influence of residual confounding on our study results, as data on some potential confounding factors were not collected from some cohort members and, thus, could not be adjusted in our study. Fourth, our participants may not fully represent each country or region. In particular, most participating cohorts came from East Asia; thus, the number of South Asian individuals is relatively small. Also, only individuals who are participants of the ACC cohorts are included in the analyses. However, we found comparable risk estimates and similar patterns of the diabetes-mortality association in each country or region when compared with the previous reports,^18,36,37^ providing some assurance for the overall external validity of our findings.

## Conclusions

The diabetes epidemic in Asia is expected to continue to accelerate; thus, many Asian individuals will live with diabetes and its complications. Diabetes substantially increases the risk of premature death from several diseases among Asian populations, and the mortality burden associated with diabetes is particularly evident in women and patients with early-onset disease. Our findings suggest the urgent need for developing diabetes management programs that are tailored to Asian populations and the subsequent strong implementation of these programs in Asia.

## Notes

Supplement. **eAppendix. **Descriptions of Participating Cohorts **eReferences** **eTable 1.** *ICD-9* and *ICD-10* Codes for Causes of Death **eTable 2.** Number of Cause-Specific Deaths Among Patients With Diabetes vs in Non-Diabetic Populations: Stratified by Individual Characteristics in Asian Populations **eTable 3.** All-Cause and Cause-Specific Mortality Associated With Diabetes: Sensitivity Analyses Excluding Subjects With a History of Cardiovascular Diseases and Any Cancers at Baseline **eTable 4.** Hazard Ratios (95% CIs) for All-Cause and Cause-Specific Mortality Associated With Diabetes: Sensitivity Analyses Excluding One Country at a Time **eFigure.** All-Cause Mortality Associated With Diabetes in Each Participating Cohort
