# Supplementary material for: Association of Diabetes With All-Cause and Cause-Specific Mortality in Asia: A Pooled Analysis of More Than 1 Million Participants
Source: JAMA Netw Open. 2019 Apr 19;2(4):e192696. doi: 10.1001/jamanetworkopen.2019.2696 (PMC6481439; doi:10.1001/jamanetworkopen.2019.2696)
Supplement: Supplement. — eAppendix. Descriptions of Participating Cohorts eReferences eTable 1. ICD-9 and ICD-10 Codes for Causes of Death eTable 2. Number of Cause-Specific Deaths Among Patients With Diabetes vs in Non-Diabetic Populations: Stratified by Individual Characteristics in Asian Populations eTable 3. All-Cause and Cause-Specific Mortality Associated With Diabetes: Sensitivity Analyses Excluding Subjects With a History of Cardiovascular Diseases and Any Cancers at Baseline eTable 4. Hazard Ratios (95% CIs) for All-Cause and Cause-Specific Mortality Associated With Diabetes: Sensitivity Analyses Excluding One Country at a Time eFigure. All-Cause Mortality Associated With Diabetes in Each Participating Cohort [file jamanetwopen-2-e192696-s001.pdf]

Supplementary Online Content

Yang JJ, Yu D, Wen W, et al. Association of diabetes with all-cause and cause-specific mortality in Asia: a pooled analysis of more than 1 million participants. *JAMA Netw Open*. 2019;2(4):e192696. doi:10.1001/jamanetworkopen.2019.2696

eAppendix. Descriptions of Participating Cohorts

eReferences

eTable 1. *ICD-9* and *ICD-10* Codes for Causes of Death

eTable 2. Number of Cause-Specific Deaths Among Patients With Diabetes vs in Non-Diabetic Populations: Stratified by Individual Characteristics in Asian Populations

eTable 3. All-Cause and Cause-Specific Mortality Associated With Diabetes: Sensitivity Analyses Excluding Subjects With a History of Cardiovascular Diseases and Any Cancers at Baseline

eTable 4. Hazard Ratios (95% CIs) for All-Cause and Cause-Specific Mortality Associated With Diabetes: Sensitivity Analyses Excluding One Country at a Time

eFigure. All-Cause Mortality Associated With Diabetes in Each Participating Cohort

This supplementary material has been provided by the authors to give readers additional information about their work.

© 2019 Yang JJ et al. *JAMA Network Open.*

eAppendix. Descriptions of Participating Cohorts

China National Hypertension Survey Epidemiology Follow-up Study [1] - This study is based on the China National Hypertension Survey in 1991, which included a nationally representative sample of the general population from all 30 provinces, autonomous regions, and municipalities of mainland China. Between 1999-2000, the China National Hypertension Survey Epidemiology Follow-up Study was conducted in 17 provinces. Using a standard questionnaire, baseline information was collected at one clinic visit by trained physicians and nurses. In-person follow-up interviews for all study participants were conducted to ascertain vital status and disease incidence. To identify cases for cardiovascular diseases and renal disorders, medical records and death certificates were reviewed. The follow-up rate was 95% over the first eight years—follow-up ended in 2000.

Shanghai Cohort Study [2] - Between January 1, 1986, and September 30, 1989, a total of 18,244 men who were aged 45-64 and had no history of cancer were recruited in Shanghai areas. During in-person interviews using a structured questionnaire, baseline information and biological samples were collected. Via data linkages to the Shanghai Cancer Registry and Shanghai Municipal Vital Statistics, as well as in-person visits, disease incidence (i.e., cancer, cardiovascular diseases, etc.) and death, were ascertained among study populations.

Shanghai Men's Health Study [3] - Between April 2002 and June 2006, a total of 61,504 cancer-free Chinese men aged 40-74 were enrolled in seven Shanghai communities. Using a validated questionnaire asking about lifestyle and dietary factors, baseline and follow-up surveys were conducted. At baseline, blood and urine samples were obtained from volunteers. Vital status and disease outcomes were regularly updated by data linkages and repeated follow-up surveys. This study is still ongoing.

Shanghai Women's Health Study [4] - Between 1997 and 2000, a total of 74,942 women aged 40-70 were enrolled in Shanghai communities, with a 92% response rate. At the baseline survey, all participants completed detailed baseline questionnaires asking dietary and lifestyle information and anthropometrics. About 88% of cohort members donated urine and blood samples. Study populations were followed via biennial in-person recontact and periodic linkage to cancer and vital statistic registries. This study is still ongoing.

Linxian General Population Trial Cohort [5] - In 1985, residents of four northern communes in Linxian, a rural county in Henan Province, were invited to participate in the study. Among total residents, 29,584 men and women, aged 40-69 and without history of cancer or debilitating disease, were randomly assigned in the initial nutrient trial. All participants were interviewed for lifestyle, medical history, family history, and diet habits and donated blood sample. Supplements (daily supplements of one or more of four vitamin and mineral combinations) were distributed in coded pill bottles from March 1, 1986, to May 1, 1991. Health outcomes were ascertained through medical review by the panel of American and Chinese experts or senior Chinese diagnosticians from Beijing. Repeated follow-up surveys were also conducted in 1991, 1996, and 1999.

Three Prefecture Cohort Study Aichi [6] - In 1985, residents of the Aichi Prefecture (Nagoya City and Inuyama City), aged ≥40 years, were invited to participate in the study. Self-administered questionnaires in sealed envelopes were distributed to all residents aged ≥40, and a total of 33,538 responded to questionnaires (response rate: ~93%). In cases of duplication or not providing basic information, some subjects were excluded from the cohort. Study participants were followed-up for death and cancer incidence through record linkage to residence certificates, death certificates, and local cancer registry data. This study was terminated in 2000.

Ibaraki Prefectural Health Study [7] - In 1993, the Ibaraki prefectural government initiated a community-based cohort study including 98,196 individuals (33,414 men and 64,782 women) aged 40-79. All study populations underwent an annual health checkup in 1993 and followed-up to ascertain deaths. If

© 2019 Yang JJ et al. *JAMA Network Open.*

participants moved out from the Ibaraki prefectural, they were treated as censored. A systematic review of death certificates and resident registrations were conducted; and the validation study showed high sensitivity and specificity of this method.

Japan Collaborative Cohort Study [8] **-** A total of 110,585 participants (46,395 men and 64,190 women) from the 45 areas of Japan were included in this study. Baseline information was collected from 1988 through 1990, using a self-administered questionnaire. Investigators reviewed the population registry information of survivors. This study was terminated at the end of 2009: a total of 27,410 deaths were identified during the median follow-up of 18 years. The main cause of death was cancer and circulatory diseases.

The Japan Public Health Center-based Prospective Study [9] **-** Based on 11 public health center areas in Japan, this study was launched in 1990 (JPHC I) and 1993 (JPHC II). A total of 140,420 subjects aged 40-59 were enrolled with a self-administered questionnaire survey. Repeated follow-up surveys were also conducted after five and ten years. Study participants were followed-up for the incidence of cancer and cardiovascular diseases and death by data linkage to cancer and residential registries. This study is still ongoing.

Three Prefecture Cohort Study Miyagi [10] **-** In 1984, residents of the Miyagi Prefecture (Sendai City and Wakuya/Tajiri Town), aged ≥40 years, were invited to participate in the study. Self-administered questionnaires in sealed envelopes were distributed to all residents aged ≥40, and a total of 31,769 responded to the questionnaires (response rate: ~97%). In cases of duplication or not providing basic information, some subjects were excluded from the cohort. Study participants were followed for death and cancer incidence through record linkage to residence certificates, death certificates, and local cancer registry data. This study was terminated in 1999.

Miyagi Cohort Study [11] **-** In 1990, this study was initiated in 14 municipalities of Miyagi Prefecture, Japan. A total of 47,605 residents aged 40-64 (coverage rate 91.7%: 13,992 men and 17,353 women) participated in this study. Two self-administered questionnaires regarding lifestyle and personality were used to collect baseline information. Through data linkage to cancer and death registries, study participants were followed-up for cancer and death. This study is still ongoing.

Ohsaki National Health Insurance Cohort Study [12] **-** In 1995, subjects who were National Health Insurance beneficiaries and received care at the Ohsaki Public Health Center were enrolled in this study. A total of 51,253 men and women aged 40-79 were recruited. Participants were regularly followed-up for death and other diseases via reviews of death certificates and National Health Insurance files. For cancer incidence, data linkage to the local cancer registry was conducted. This study is still ongoing.

Life Span Study [13] **-** This study consists of 120,000 persons including atomic bomb survivors, 94,000 of whom were in the city at the time of the bombing. Another 26,000 were age- and sex-matched residents who were not in Hiroshima or Nagasaki at the time of the bombing. This cohort was established based on the 1950 Japanese national census. Participants were followed-up for mortality and cancer incidence. Lifestyle information was collected via a clinical sub-study and mailed questionnaires. For this unique cohort, participants were restricted to those who were exposed to less than 0.1 grays of bomb radiation. This study is still ongoing.

Takayama study [14] **-** This study was initiated on September 1, 1992. At the baseline survey, a total of 31,552 Takayama residents, aged ≥35 years (14,427 men and 17,125 women, representing 85.3% of the total population), completed a self-administered questionnaire. Study participants were followed-up for cancer diagnosis, death, or emigration. The most recent follow-up was completed in 2008.

Korea Multi-Center Cancer Cohort [15] **-** Between 1993 and 2004, 19,688 men and women over 18 years of age were recruited from four areas (Haman, Choongju, Uljin, and Pohang) of the Republic of Korea.

© 2019 Yang JJ et al. *JAMA Network Open.*

Baseline information on general lifestyle, physical activity, diet, reproductive factors, and others were collected by direct interview. Blood (plasma, or serum buffy coat, packed erythrocytes) and urine samples were also obtained. Based on data linkage with the national cancer registry, death-certificate system, and health-insurance databases, study participants were regularly followed-up. The final updates were done to identify all deaths that occurred until the end of 2014 and cancers that occurred until the end of 2013. This study is still ongoing.

Seoul Male Cohort Study [16] - This study was initiated in 1992, with an enrollment of a total of 29,918 men aged 40-59. Baseline information was collected by self-administered questionnaires from 14,533 participants. Death certificates from the National Statistics Office were used to ascertain vital status. Study participants were followed-up from January 1, 1993, to December 31, 2008.

Singapore Chinese Health Study [17] - This study aims to investigate the role of diet and genetic factors in cancer etiology. Between 1993 and 1999, a total of 63,257 men and women, aged 45-74, were recruited for this cohort. At the recruitment, participants were interviewed using staff-administered questionnaires including a validated Food Frequency Questionnaire. Biologic samples were also obtained from consenting cohort members. Study participants were regularly followed-up for cancer incidence and mortality via record linkages to local cancer and vital status registries.

Community-Based Cancer Screening Project [18] - From January 1991 to December 1992, a total of 23,820 individuals, aged 30-65, were recruited from seven townships in Taiwan. Using structured questionnaires, baseline and follow-up information were administered by trained staff. Participants were followed-up for cancer incidence and death through health examination, medical record review, and data linkage to the national cancer registry and death certification systems.

CardioVascular Disease risk FACtor Two-township Study [19] - Between 1990-1993, a total of 5,160 adults were recruited from Chu-Don, a Hakka community in northwest Taiwan and from Pu-Tze, a Fukien community in southern Taiwan. In each of the two communities, five villages with >1,000 people or with a population density >200 per square kilometer were randomly selected for this study. Follow-up is conducted every three years using death certificates provided by the Department of Health in Taiwan.

Health Effects for Arsenic Longitudinal Study Bangladesh [20] - Between 2000 and 2002, 11,746 married men and women, aged ≥18, were recruited for the study (response rate=97.5%). At the baseline survey, data on demographic and lifestyle factors were collected. Using an automatic sphygmomanometer, blood pressure was measured by trained clinicians. Study participants were actively followed-up every two years with in-person visits that include a physical examination and urine collection. This study is still ongoing.

Mumbai Cohort Study [21] - This study recruited participants in two phases, 1991-1994 and 1994-1996. Participants were residents of Mumbai and ≥35 years of age. About 150,000 subjects were interviewed at baseline. To ascertain participants' vital status, an active house-to-house follow-up was done after an average of 5.5 years. Data on cause of death was obtained from the local death registry. Cancer incidence was ascertained by data linkage to cancer registry databases. This study is still ongoing.

© 2019 Yang JJ et al. *JAMA Network Open.*

eReferences.

1. He J, Gu D, Chen J, Wu X, Kelly TN, Huang JF, Chen JC, Chen CS, Bazzano LA, Reynolds K, Whelton PK, Klag MJ. Premature deaths attributable to blood pressure in China: a prospective cohort study. Lancet.2009;374(9703):1765-72.
2. Moy KA, Yuan JM, Chung FL, Wang XL, Van Den Berg D, Wang R, Gao YT, Yu MC.Isothiocyanates, glutathione S-transferase M1 and T1 polymorphisms and gastric cancer risk: a prospective study of men in Shanghai, China. Int J Cancer. 2009;125(11):2652-9.
3. Shu XO, Li H, Yang G, Gao J, Cai H, Takata Y, Zheng W, Xiang YB. Cohort Profile: The Shanghai Men's Health Study. Int J Epidemiol. 2015;44(3):810-8.
4. Zheng W, Chow WH, Yang G, Jin F, Rothman N, Blair A, Li HL, Wen W, Ji BT, Li Q, Shu XO, Gao YT. The Shanghai Women's Health Study: rationale, study design, and baseline characteristics. Am J Epidemiol. 2005;162(11):1123-31.
5. Qiao YL, Dawsey SM, Kamangar F, Fan JH, Abnet CC, Sun XD, Johnson LL, Gail MH, Dong ZW, Yu B, Mark SD, Taylor PR. Total and cancer mortality after supplementation with vitamins and minerals: follow-up of the Linxian General Population Nutrition Intervention Trial. J Natl Cancer Inst. 2009;101(7):507-18.
6. Sado J, Kitamura T, Kitamura Y, Zha L, Liu R, Sobue T, Nishino Y, Tanaka H, Nakayama T, Tsuji I, Ito H, Suzuki T, Katanoda K, Tominaga S; Three-Prefecture Cohort Study Group. Rationale, design, and profile of the Three-Prefecture Cohort in Japan: A 15-year follow-up. J Epidemiol. 2017;27(4):193-199.
7. Noda H, Iso H, Irie F, Sairenchi T, Ohtaka E, Doi M, Izumi Y, Ohta H. Low-density lipoprotein cholesterol concentrations and death due to intraparenchymal hemorrhage: the Ibaraki Prefectural Health Study. Circulation. 2009;119(16):2136-45.
8. Tamakoshi A, Ozasa K, Fujino Y, Suzuki K, Sakata K, Mori M, Kikuchi S, Iso H; JACC Study Group, Sakauchi F, Motohashi Y, Tsuji I, Nakamura Y, Mikami H, Kurosawa M, Hoshiyama Y, Tanabe N, Tamakoshi K, Wakai K, Tokudome S, Hashimoto S, Wada Y, Kawamura T, Watanabe Y, Miki T, Date C, Kurozawa Y, Yoshimura T, Shibata A, Okamoto N, Shio H. Cohort profile of the Japan Collaborative Cohort Study at final follow-up. J Epidemiol. 2013;23(3):227-32.
9. Watanabe S, Tsugane S, Sobue T, Konishi M, Baba S. Study design and organization of the JPHC study. Japan Public Health Center-based Prospective Study on Cancer and Cardiovascular Diseases. J Epidemiol. 2001;11(6 Suppl):S3-7.
10. Sado J, Kitamura T, Kitamura Y, Zha L, Liu R, Sobue T, Nishino Y, Tanaka H, Nakayama T, Tsuji I, Ito H, Suzuki T, Katanoda K, Tominaga S; Three-Prefecture Cohort Study Group. Rationale, design, and profile of the Three-Prefecture Cohort in Japan: A 15-year follow-up. J Epidemiol. 2017;27(4):193-199.
11. Tsuji I, Nishino Y, Tsubono Y, Suzuki Y, Hozawa A, Nakaya N, Fujita K, Kuriyama S, Shibuya D, Fukao A, Hisamichi S. Follow-up and mortality profiles in the Miyagi Cohort Study. J Epidemiol. 2004;14 Suppl 1:S2-6.
12. Tsuji I, Nishino Y, Ohkubo T, Kuwahara A, Ogawa K, Watanabe Y, Tsubono Y, Bando T, Kanemura S, Izumi Y, Sasaki A, Fukao A, Nishikori M, Hisamichi S. A prospective cohort study on National Health Insurance beneficiaries in Ohsaki, Miyagi Prefecture, Japan: study design, profiles of the subjects and medical cost during the first year. J Epidemiol. 1998;8(5):258-63.
13. Ozasa K, Shimizu Y, Suyama A, Kasagi F, Soda M, Grant EJ, Sakata R, Sugiyama H, Kodama K. Studies of the mortality of atomic bomb survivors, Report 14, 1950-2003: an overview of cancer and noncancer diseases. Radiat Res. 2012;177(3):229-43.
14. Tamura T, Wada K, Konishi K, Goto Y, Mizuta F, Koda S, Hori A, Tanabashi S, Matsushita S, Tokimitsu N, Nagata C. Coffee, Green Tea, and Caffeine Intake and Liver Cancer Risk: A Prospective Cohort Study. Nutr Cancer. 2018 Nov 20:1-7.
15. Yoo KY, Shin HR, Chang SH, Lee KS, Park SK, Kang D, Lee DH. Korean Multi-center Cancer Cohort Study including a Biological Materials Bank (KMCC-I). Asian Pac J Cancer Prev. 2002;3(1):85-92.

© 2019 Yang JJ et al. *JAMA Network Open.*

16. Rhee CW, Kim JY, Park BJ, Li ZM, Ahn YO. Impact of individual and combined health behaviors on all causes of premature mortality among middle aged men in Korea: the Seoul Male Cohort Study. J Prev Med Public Health. 2012;45(1):14-20.
17. Hankin JH, Stram DO, Arakawa K, Park S, Low SH, Lee HP, Yu MC. Singapore Chinese Health Study: development, validation, and calibration of the quantitative food frequency questionnaire. Nutr Cancer. 2001;39(2):187-95.
18. Chen CJ, Yang HI, Su J, Jen CL, You SL, Lu SN, Huang GT, Iloeje UH; REVEAL-HBV Study Group. Risk of hepatocellular carcinoma across a biological gradient of serum hepatitis B virus DNA level. JAMA. 2006; 295(1):65-73.
19. Chuang SY, Bai CH, Chen WH, Lien LM, Pan WH. Fibrinogen independently predicts the development of ischemic stroke in a Taiwanese population: CVDFACTS study. Stroke. 2009;40(5):1578-84.
20. Chen Y, Wu F, Liu M, Parvez F, Slavkovich V, Eunus M, Ahmed A, Argos M, Islam T, Rakibuz-Zaman M, Hasan R, Sarwar G, Levy D, Graziano J, Ahsan H. A prospective study of arsenic exposure, arsenic methylation capacity, and risk of cardiovascular disease in Bangladesh. Environ Health Perspect. 2013;121(7):832-8.
21. Pednekar MS, Hakama M, Hebert JR, Gupta PC. Association of body mass index with all-cause and cause-specific mortality: findings from a prospective cohort study in Mumbai (Bombay), India. Int J Epidemiol. 2008;37(3):524-35.

© 2019 Yang JJ et al. *JAMA Network Open.*

eTable 1. ICD-9 and ICD-10 codes for causes of death

	ICD-9	ICD-10
**Death from all-causes**	001-E999	A00-Y98
**Cardiovascular diseases**	390-459	I00-I99
Coronary heart diseases	410-414	I20-I25
Stroke	430-438	I60-I69
Ischemic stroke	434	I63
Hemorrhagic stroke	430-432	I60-I62
**Endocrine, nutritional and metabolic diseases**	240-279	E00-E90
Diabetes mellitus	249-250	E10-E14
**Cancer**	140-209	C00-C99
Lung	162	C34
Breast	174-175	C50
Colorectal	153-154	C18-C21
Stomach	151	C16
Liver	155	C22
Pancreas	157	C25
Gallbladder	156	C23-C24
Female reproductive organs [a]	179-184	C51-C58
**Renal diseases**	580-629	N00-N99
Kidney failure	584-586	N17-N19
**Digestive system diseases**	520-579	K00-K93
Liver disorders [b]	570-573	K70-K77
Gallbladder, biliary tract, and pancreas	574-577	K80-K87
Stomach, esophagus, and duodenum	530-539	K20-K31
**Respiratory diseases**	460-519	J00-J99
Pneumonia	480-487	J12-J18
Asthma	493	J45-J46
Chronic obstructive pulmonary disease	490-492, 494-496	J40-J44, J47
**Infectious diseases**	001-139	A00-B99
Tuberculosis	010-018	A15-A19
**Other known diseases**		
Mental disorder	290-319	F00-F99
Nervous system disorder	520-529	G00-G99

ICD-9, the 9th revision of the International Statistical Classification of Diseases and Related Health Problems; ICD-10, the 10th revision of the International Statistical Classification of Diseases and Related Health Problems;

[a] Malignant neoplasms of vagina, cervix uteri, corpus uteri, uterus, ovary, vulva, and other female genital organs

[b] Liver cirrhosis, hepatic failure, chronic hepatitis, fibrosis, alcoholic/toxic liver disease, and other kinds of liver disorders

© 2019 Yang JJ et al. *JAMA Network Open.*

eTable 2. Number of Cause-specific Deaths among Patients with Diabetes vs. Non-Diabetic Populations: stratified by individual characteristics in Asian populations

	No. of Deaths in Patients with Diabetes vs. in Non-Diabetics Populations									
	**CVD**	**CHD**	**I-Stroke**	**H-Stroke**	**DM**	**Cancer**	**Renal Disease**	**Digestive Disease**	**Respiratory Disease**	**Infectious Disease**
**Total populations**	4,147/37,523	1,471/9,107	576/4,815	470/6,433	1,456/1,165	2,861/39,571	413/2,315	437/4,296	1,149/12,061	200/2,503
**Sex**										
Men	2,198/21,216	806/5,704	277/2,714	259/3,447	708/536	1,885/24,593	191/1,181	275/2,563	766/7,790	123/1,504
Women	1,949/16,307	665/3,403	299/2,101	211/2,986	748/629	976/14,978	222/1,134	162/1,733	383/4,271	77/999
**Age at baseline, years**										
< 50	323/3,800	103/942	31/282	70/1,046	125/187	286/7,409	34/220	72/867	77/857	20/437
50-59	991/8,920	386/2,343	120/998	115/1,678	353/378	863/13,026	115/558	131/1,241	249/2,653	60/732
60-69	1,828/14,796	667/3,684	257/2,015	201/2,243	687/467	1,213/14,164	165/912	166/1,475	486/4,820	80/909
≥ 70	1,005/10,007	315/2,138	168/1,520	84/1,466	291/133	499/4,972	99/625	68/713	337/3,731	40/425
**Educational attainment** [a]										
≤ Primary school	2,008/18,987	787/4,372	272/2,472	177/3,066	789/695	1,164/17,562	210/1,168	183/2,157	519/5,645	76/1,050
Secondary school	954/8,385	300/1,995	144/1,036	140/1,739	290/198	770/10,204	92/467	122/1,013	285/2,775	46/452
Trade/technical school	280/1,904	92/558	18/131	54/458	83/62	206/2,550	17/116	26/215	55/565	18/175
≥ University graduation	227/2,292	81/848	18/183	29/286	79/80	150/2,217	15/128	23/214	52/767	9/331
**BMI, kg/m^2**										
<18.5	191/4,212	59/863	15/477	18/674	88/46	136/3,014	25/232	27/462	84/2,111	16/613
18.5 - 22.9	1,548/16,751	537/3,934	203/2,111	204/2,945	534/382	1,132/18,745	157/994	174/2,068	508/5,925	87/1,155
23.0 - 24.9	1,100/7,823	402/2,044	156/1,027	114/1,292	348/251	713/8,930	103/505	108/832	284/2,206	47/379
≥ 25.0	1,308/8,737	473/2,266	202/1,200	134/1,522	486/486	880/8,882	128/584	128/934	273/1,819	50/356
**Smoking status - Men**										
Never	546/5,560	229/1,560	58/721	52/869	224/160	307/4,523	54/303	46/611	147/1,566	26/448
Former	542/3,664	203/1,018	72/471	65/548	140/72	442/3,972	44/226	43/346	254/1,857	38/254
Current	1,110/11,992	374/3,126	147/1,522	142/2,030	344/304	1,136/16,098	93/652	186/1,606	365/4,367	59/802
**Smoking status - Women**										
Never	1,664/13,923	560/2,849	254/1,816	175/2,534	651/573	844/13,104	192/994	128/1,512	322/3,586	69/910
Former/Current [b]	285/2,384	105/554	45/285	36/452	97/56	132/1,874	30/140	34/221	61/685	8/89

CVD, cardiovascular disease; CHD, coronary heart disease; I-Stroke, ischemic stroke; H-Stroke, hemorrhagic stroke; DM, diabetes mellitus;

[a] Cohorts (3 Prefecture Aichi / Miyagi Study, Ibaraki Prefectural Health Study, Japan Public Health Center-based prospective Study2, Korean Multi-center Cancer Cohort Study) without information on education were not included in the analysis

[b] Former- and current-smokers were combined to improve the stability of point estimates

© 2019 Yang JJ et al. *JAMA Network Open.*

eTable 3. All-cause and cause-specific mortality associated with diabetes: Sensitivity analyses excluding subjects with a history of cardiovascular diseases and any cancers at baseline

Individual Characteristics / Death Causes	Diabetes, *n*		No Diabetes, *n*		HR (95% CI) [a]	*P* interaction
	Participants	Death	Participants	Death		
**All-cause Mortality**						
**Total population**	36,901	10,417	918,672	125,142	1.88 (1.72-2.04)	NA
**Sex**						
Men	20,762	6,146	441,964	73,311	1.74 (1.60-1.88)	<0.01
Women	16,139	4,271	476,708	51,831	2.09 (1.88-2.33)	
**Age at baseline, years**						
< 50	7,065	1,054	354,313	18,502	2.45 (2.07-2.90)	<0.01
50-59	12,534	2,772	292,276	34,991	1.99 (1.78-2.23)	
60-69	12,859	4,354	205,860	46,230	1.87 (1.70-2.05)	
≥ 70	4,443	2,237	66,223	25,419	1.49 (1.38-1.61)	
**Educational attainment** [b]						
≤ Primary school	13,275	4,446	311,656	60,180	1.97 (1.73-2.26)	0.95
Secondary school	9,405	2,409	219,143	25,062	1.96 (1.71-2.24)	
Trade/technical school	3,247	576	95,615	5,860	1.68 (1.53-1.84)	
≥ University graduation	3,016	549	98,927	7,440	1.57 (1.37-1.80)	
**BMI, kg/m^2**						
<18.5	1,366	616	67,481	13,667	1.89 (1.62-2.22)	0.18
18.5 - 22.9	13,258	4,155	412,175	60,419	1.97 (1.77-2.19)	
23.0 - 24.9	9,549	2,550	213,490	25,556	1.83 (1.66-2.03)	
≥ 25.0	12,728	3,096	225,526	25,500	1.84 (1.68-2.01)	
**Smoking status - Men**						
Never	6,109	1,322	145,882	17,209	1.81 (1.60-2.04)	0.45
Former	4,287	1,330	61,413	11,229	1.60 (1.45-1.76)	
Current	10,366	3,494	234,669	44,873	1.71 (1.58-1.85)	
**Smoking status - Women**						
Never	14,477	3,662	441,793	45,817	2.14 (1.92-2.38)	0.01
Former	362	133	5,075	907	2.04 (1.56-2.66)	
Current	1,300	476	29,840	5,107	1.81 (1.57-2.08)	
**Cause-specific Mortality**						
**Cardiovascular disease**		3,312		33,233	2.00 (1.75-2.30)	NA
**Coronary heart disease**		1,164		8,016	2.65 (2.25-3.12)	NA
**Ischemic stroke**		457		4,182	2.17 (1.84-2.55)	NA
**Hemorrhagic stroke**		377		5,727	1.37 (1.16-1.61)	NA
**Diabetes mellitus**		1,216		1,067	22.9 (18.5-28.4)	NA
**Cancer**		2,448		36,846	1.20 (1.11-1.29)	NA
**Renal disease**		345		2,096	3.05 (2.45-3.79)	NA
**Digestive disease**		375		4,048	1.92 (1.71-2.15)	NA
**Respiratory disease**		978		11,034	1.46 (1.29-1.65)	NA
**Infectious disease**		177		2,377	1.94 (1.55-2.45)	NA

HR = Hazard ratio; 95% CI = 95% confidence interval; NA = not available

[a] Adjusted for age, sex, smoking status, education, marital status, rural/urban residence, and obesity status; and stratified by birth year (5-year groups) and enrollment year (5-year groups)

[b] Cohorts (3 Prefecture Aichi / Miyagi Study, Ibaraki Prefectural Health Study, Japan Public Health Center-based prospective Study2, Korean Multi-center Cancer Cohort Study) without information on education were not included in the analysis

© 2019 Yang JJ et al. *JAMA Network Open.*

eTable 4. All-cause and cause-specific mortality associated with diabetes: Sensitivity analyses excluding one country at a time

Death Causes	Adjusted HR (95% CI) [a]						
	Excluding China	Excluding Japan	Excluding Korea	Excluding Singapore	Excluding Taiwan	Excluding Bangladesh	Excluding India
**All-causes**	1.81 (1.65-1.99)	2.10 (1.88-2.35)	1.93 (1.77-2.09)	1.86 (1.73-2.01)	1.82 (1.68-1.96)	1.88 (1.73-2.03)	1.90 (1.75-2.06)
**Cardiovascular disease**	2.09 (1.78-2.45)	2.13 (1.73-2.63)	2.03 (1.78-2.33)	1.94 (1.74-2.17)	1.92 (1.68-2.20)	1.97 (1.72-2.26)	2.01 (1.75-2.30)
**Coronary heart disease**	2.63 (2.19-3.16)	2.63 (2.02-3.42)	2.63 (2.23-3.10)	2.48 (2.16-2.85)	2.44 (2.07-2.88)	2.59 (2.20-3.05)	2.61 (2.21-3.08)
**Ischemic stroke**	2.15 (1.79-2.59)	2.21 (1.81-2.69)	2.16 (1.84-2.53)	2.15 (1.83-2.53)	2.12 (1.81-2.49)	2.15 (1.85-2.51)	2.17 (1.85-2.53)
**Hemorrhagic stroke**	1.39 (1.17-1.63)	1.39 (1.01-1.91)	1.39 (1.19-1.64)	1.39 (1.18-1.64)	1.33 (1.13-1.56)	1.38 (1.17-1.62)	1.38 (1.17-1.63)
**Diabetes mellitus**	20.5 (16.4-25.6)	20.7 (15.1-28.4)	23.4 (18.7-29.4)	23.2 (18.5-29.1)	24.3 (19.4-30.5)	22.4 (18.2-27.6)	24.1 (19.7-29.4)
**Cancer**	1.23 (1.17-1.30)	1.13 (0.97-1.31)	1.23 (1.15-1.31)	1.20 (1.12-1.29)	1.20 (1.12-1.28)	1.21 (1.13-1.29)	1.21 (1.13-1.29)
**Renal disease**	3.19 (2.51-4.04)	3.61 (2.54-5.13)	3.07 (2.48-3.80)	3.09 (2.46-3.90)	2.74 (2.33-3.23)	3.04 (2.47-3.74)	3.11 (2.52-3.84)
**Digestive disease**	2.08 (1.80-2.40)	1.83 (1.43-2.33)	1.99 (1.73-2.28)	1.91 (1.69-2.17)	1.98 (1.73-2.26)	1.97 (1.72-2.26)	1.98 (1.73-2.27)
**Respiratory disease**	1.53 (1.36-1.72)	1.21 (0.94-1.56)	1.46 (1.30-1.65)	1.42 (1.27-1.59)	1.46 (1.30-1.64)	1.46 (1.30-1.64)	1.46 (1.29-1.64)
**Infectious disease**	1.85 (1.49-2.29)	2.17 (1.54-3.05)	1.97 (1.59-2.46)	1.93 (1.54-2.42)	1.93 (1.56-2.39)	1.96 (1.59-2.41)	2.02 (1.64-2.49)

HR = Hazard ratio; 95% CI = 95% confidence interval; NA = not available

[a] Adjusted for age, sex, smoking status, education, marital status, rural/urban residence, and obesity status; and stratified by birth year (5-year groups) and enrollment year (5-year groups)

[b] Cohorts (3 Prefecture Aichi / Miyagi Study, Ibaraki Prefectural Health Study, Japan Public Health Center-based prospective Study2, Korean Multi-center Cancer Cohort Study) without information on education were not included in the analysis

© 2019 Yang JJ et al. *JAMA Network Open.*

eFigure. **All-cause mortality associated with diabetes in each participating cohort**

Participating Cohorts	Adjusted HR [95% CI]	Weight (%)
CHEFS - Rural	2.97 [2.34 – 3.78]	3.46
CHEFS - Urban	1.82 [1.63 – 2.02]	4.67
SCS	2.55 [2.13 – 3.05]	4.03
SMHS	1.74 [1.60 – 1.90]	4.82
SWHS	2.14 [2.00 – 2.29]	4.92
LINXIAN	2.00 [1.33 – 3.01]	2.14
3Pref Aichi	1.93 [1.71 – 2.17]	4.58
IPHS	1.81 [1.67 – 1.96]	4.85
JACC	1.61 [1.50 – 1.72]	4.93
JPHC1	1.79 [1.64 – 1.95]	4.82
JPHC2	1.67 [1.58 – 1.78]	4.97
3Pref Miyagi	1.58 [1.44 – 1.73]	4.78
Miyagi	1.99 [1.82 – 2.18]	4.79
Ohsaki	1.61 [1.50 – 1.72]	4.93
LSS	1.42 [1.35 – 1.49]	5.01
Takayama	1.55 [1.48 – 1.63]	5.01
KMCC	1.48 [1.25 – 1.76]	4.12
Seoul Male	1.38 [1.01 – 1.89]	2.83
SCHS	2.29 [2.17 – 2.42]	4.99
CBCSP	3.09 [2.69 – 3.55]	4.41
CVDFACTS	2.65 [2.11 – 3.31]	3.61
HEALS	2.29 [1.68 – 3.11]	2.86
Mumbai	1.67 [1.47 – 1.91]	4.47
Overall (*I*-squared = 94.0%, *p* <0.01)	1.89 [1.74 – 2.04]	100.0

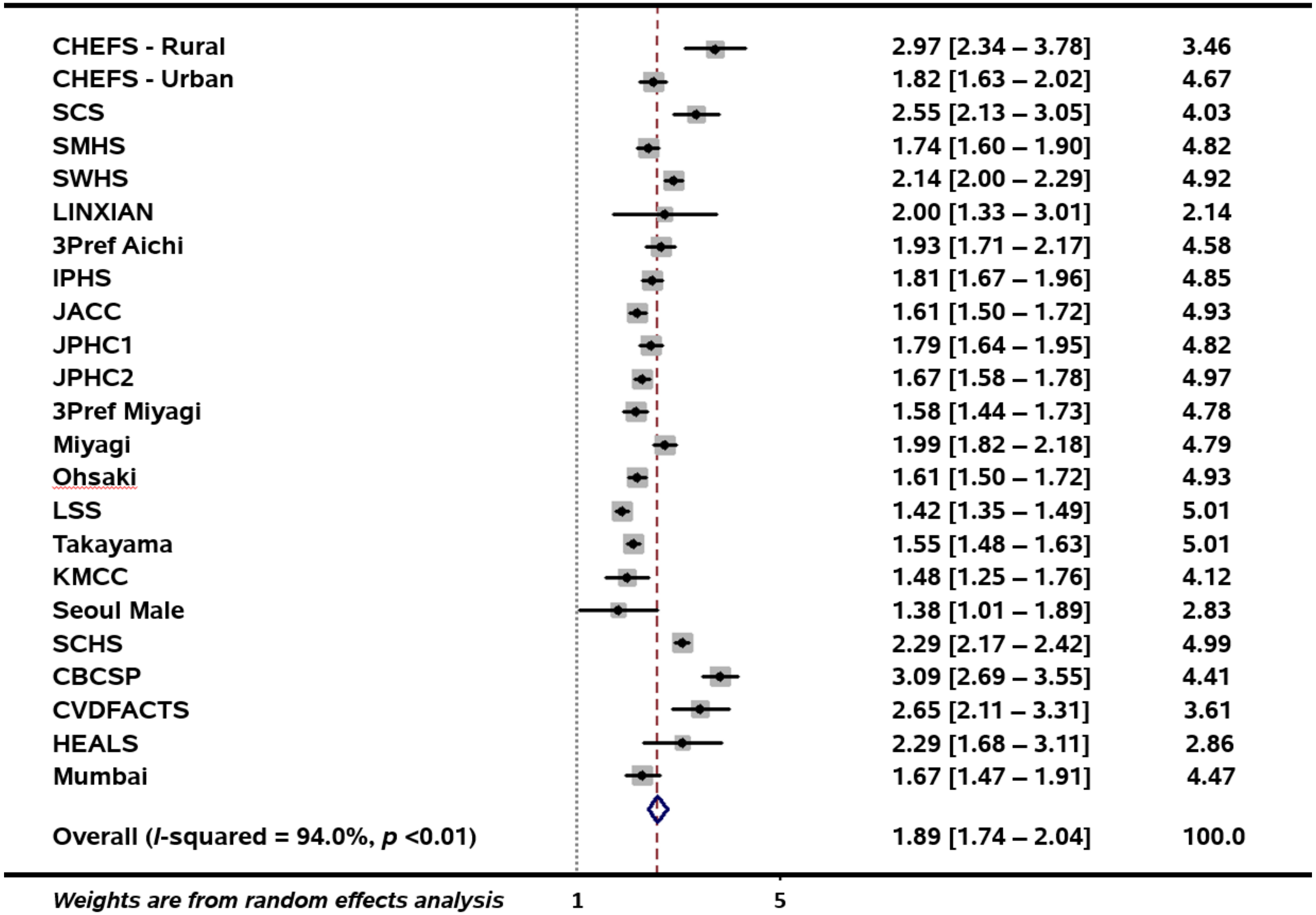

Weights are from random effects analysis

Hazard ratios (HRs) and 95% CIs (95% confidence intervals) were adjusted for age, sex, smoking status, smoking pack-years, education, marital status, rural/urban residence, and obesity status; and stratified by birth year (5-year groups) and enrollment year (5-year groups). The CHEFS, the representative sample of the general population from all 30 provinces in China, was divided by rural and urban areas due to a substantial difference in lifestyle, accessibility to medical services, and diagnosis/treatment for diabetes across China.

© 2019 Yang JJ et al. *JAMA Network Open.*
